# Notes and News

**Published:** 1888-04-21

**Authors:** 


### Notes and News.

Collected and Communicated.

Lord Justice Bowen will preside at the annual festival dinner of King's College Hospital, which will be held in the Whitehall Rooms of the Hotel Metropole, on Monday, the 30th inst.

Mr. W. Burdett-Coutts, M.P., will read a paper at the Governor's Hall of St. Thomas's Hospital, on "Contributions by Patients in Relation to the Financial Condition of London Hospitals," on Wednesday, April 25th, at eight p.m. It is expected that the paper will give rise to a good deal of discussion, and that a great many hospital managers and others interested in the subject will be present. The occasion is the usual monthly meeting of The Hospitals Association.

The Earl of Strafford presided at the annual meeting of the governors, subscribers, and friends of the Gordon Hospital, 278, Vauxhall Bridge Road, at Willis's Rooms, King Street, St. James's, on Wednesday, 18th. The report for the past year was submitted, the regulations as amended by the committee were considered, and members of committee were elected to fill vacancies.

Sir Archibald Orr-Ewing, presiding at the annual meeting of the subscribers to the Glasgow Hospital for Skin Diseases, remarked that it was surprising that the subscriptions to this institution should have amounted to only £222. It is indeed strange, and hardly consonant with the well-known generosity of the good folks of St. Mungo's city towards their charities. In concluding his speech Sir Archibald paid a well-deserved tribute to Dr. M'Call Anderson, to whom the hospital owes its origin.

Although we do not agree with the enthusiastic Teuton in Mr. Besant's last book, who protests that London depends for its greatness on the German residents in it, we think these form a sufficiently numerous and important body to justify those among them who are rich keeping a hospital for those who are poor. The German Hospital at Dalston, however, is no selfish institution, which confines its usefulness to those who hail from the Fatherland. It was stated at the anniversary dinner of the institution that of the 653,000 patients who have been treated there during its forty-three years of existence 259,000 were of English nationality. In view of this fact we are glad to learn that the hospital receives a considerable amount of support from the English community. The subscriptions received in connexion with the festival dinner amount to £4,338, and include £200 from the Emperor of Germany, £50 from the Emperor of Austria, and £50 from the Lord Mayor, who was the first in London to appeal to the public for help for the German Hospital.

A milkseller at Govan, N.B., has been fined £8 fo selling milk adulterated with 10 per cent. of water.

A new wing will shortly be opened at Berry Wood Asylum, Northampton, for the treatment of idiot children, and the Medical Superintendent is prepared to receive applications for the admission of pauper or private cases.

Mr. John Rendall, F.R.C.S., of Lymington, in his report to the Local Board on the health of that town, complains that so much delay occurs in getting patients suffering from infectious diseases into the so called "Fever Hospital," that the advantages of isolation are completely nullified. It is, indeed, very difficult to impress on parochial and other authorities that though hospitals for infectious diseases are not in constant use they should be constantly kept ready for use.

Mark Twain on one occasion speaks of giving his advice for what it is worth—or less. We modestly put the same price on the following anecdote. Sir James Paget not long ago bound up in provisional fashion the fractured leg of a man who had been run over by a cab, and then accompanied the sufferer to a hospital, where the house surgeon on duty, who did not recognise his eminent professional brother, is said to have rated him vigorously for his "clumsy interference" with the case.

The officials of the Jenny Lind Infirmary, Norwich, as well as many others in the town, are mourning the loss of Mr. Horace Turner, one of the surgeons to the institution, who died on the 27th of March, from diphtheria, caught from some of his patients at Thorpe. Although only thirty-nine years of age, Mr. Turner had won a high reputation not only for skill but for self-sacrificing attention to every case he undertook. At the last meeting of the Committee of Management of the Jenny Lind Infirmary, Mr. S. R. Master suggested that a cot in the hospital should be named "The Turner Cot" as a memento of the friend whom they had lost, and Mr. J. J. Winter moved a vote of condolence and sympathy to Mrs. Horace Turner and her family.

Two important questions were brought before the supporters of the Leicester Infirmary, at their annual meeting. One of these was the size of the existing fever house, which seems to be too small for the demands made on the accommodation. A fever house is not the sort of hospital that is always in requisition, and therefore people are apt to forget that when it is needed at all the need is great, and ought to be sufficiently met. At the beginning of 1886 there were no patients in the fever house of Leicester Infirmary, but during the year 63 were admitted, and during last year 143, of whom 18—all the house will hold—are still under treatment. If any more fever cases make their appearance, as the authorities seem to think not unlikely, there is the risk of their not being received; and isolation, the one sure preventive for the spread of infectious diseases, will be impossible. This is a matter which demands immediate attention. In such things to wait for wisdom till after the event is folly's self.

The other suggestion brought before the Leicester public is the building of a Children's Hospital, which it is unanimously considered desirable to have erected. The only difference of opinion which exists belongs to the question of a suitable site. A piece of land adjacent to the infirmary can be obtained gratis, but the general consensus of opinion was in favour of having the new hospital entirely separate from the existing one, and as one of those who most strongly advocate the latter plan is a gentleman who has promised £1,000 towards the erection of the hospital, it may be taken for granted that the "separatists," who in this matter certainly have right on their side, will gain the day.

THE following vacancies are announced this week, the amount of salary in each case being given after the name of the institution :—

**Resident Medical Officers.**

Birmingham General Dispensary. Surgeon.—£150 per annum, and £30 for cab hire.
Birmingham General Hospital. Assistant House Surgeon.—Board; no salary.
Norfolk County Asylum, Thorpe, near Norwich. Junior Assistant Medical Officer.—£100, with board.
North-West London Hospital, Kentish Town Road. Senior Resident Medical Officer.
Rotherham Hospital. Assistant House Surgeon. Board, etc.
St. Luke's Hospital. Resident Clinical Assistant.—Board, etc.

**Non-resident Medical Officers.**

Borough of Brighton. Medical Officer of Health.—£500 per annum.
Bristol Royal Infirmary. Honorary Assistant Physician.
Durham County Hospital. Honorary Surgeon.
Durham County Hospital. Honorary Surgeon-Dentist.
Liddell Provident Dispensary, Jarrow-on-Tyne. Medical Officer.—£200 per annum.
Parish of Tarbet Easter, Ross-shire, N.B. Medical Officer.—£115 per annum.
Seamen's Hospital, Greenwich. Visiting Physician.

---

THE Nottingham General Hospital is in such straits for money that the Board of Governors have been compelled to expend the sum received in legacies during last year—about £1,800—in current expenses, instead of investing it, as they would like to do. Under these circumstances they might do well to consider the suggestion made at the annual meeting by Mr. H. E. Thornton, that when beds are empty the board should be permitted to receive paying patients to fill them. Better by far to have paying patients than beds kept empty for lack of means; but it is only too likely that there are sick poor enough in Nottingham to fill the hospital to the full. Will not their richer brethren in the energetic midland town come forward in such a manner as to make this possible?

---

THE quinquennial appeal of the London Hospital ought to be trumpeted far and wide. This hospital has claims which are not local, but metropolitan, one might say national. The East-end of London is in some sense the workshop of the world, and the workers in it pursue their labours under very unfavourable conditions for the preservation of health. When they fall ill their natural refuge is the hospital in Whitechapel Road, where upwards of 104,000 patients were treated last year, at a cost of something over £50,000. It is obvious that this sum cannot be collected at the East-end, whose wealthiest inhabitants are not in affluent circumstances, and we hope that the appeal of the authorities to the rest of London for help towards the maintenance of the hospital will meet with a liberal response.

---

AN incident which recently occurred in Birmingham may do something towards discouraging that abuse of charities of which so many people complain. A child, whose parents appear to have been in sufficiently comfortable circumstances, was sent to the Children's Hospital suffering rom what seemed to be a severe cold. The temptations were cheapness in the first place; in the second, but only in the second, good medical attendance. As the severe cold turned out to be a case of scarlatina, the exposure to cold involved in the journey to the hospital was the worst possible treatment, and resulted in the child's death. An inquest was held, and the coroner spoke very severely of the conduct of the parents, who, without the sad excuse of poverty, exposed their child to such a danger. Sad as such an accident is, it may act as a salutary warning to people who, for the sake of an unworthy economy, seek medical treatment gratis.

"THE Silver Fête" which is to be held in the Royal Exhibition grounds, South Kensington, from July 11th to 14th, promises to be one of the most attractive events of the season. At the head of a long list of royal and noble patronesses stands the name of the gracious lady from whose silver wedding the festival takes its title, who will, we are sure, be anxious that no untoward event shall mar this feast of charity as the silver anniversary of her wedding day was darkened. The object for which the fête is held is to clear off a debt which now encumbers the Victoria Hospital for Children, Queen's Road, Chelsea, and with such attractions as the promoters of the fête promise there should be little difficulty in raising the £3,000 needed. The items promised include a grand costume bazaar, to be held in the Conservatory of the Albert Hall; a scene of revels, including maypole dances, lawn tennis tournaments, and amusements of all sorts; a temple of music, where distinguished artistes will perform; and an "Eldorado," where most of the leading lights of dramatic art have promised to appear. With attractions thus suited to all tastes the success of the fête should be sure.

---

THE Congress of American physicians and surgeons, which is to take place at Washington on the evenings of the 18th, 19th, and 20th September, will be a meeting of no common interest. It will be a grand reunion of the men most distinguished in medicine and surgery throughout the United States, and of the chief associations formed throughout America for the study of special developments of disease. It is proposed that all these associations should meet at Washington at the dates given above, and, preserving complete independence, hold their meetings, read papers, etc., during the day, uniting every evening in the general Congress, where very important papers will be read by Drs. Reginald H. Fitz, Nicholas Senn, Charles K. Mills, and Roswell Park—gentlemen whose renown has crossed the Atlantic. The closing address will be given by the President, Dr. John S. Billings, of the U.S. Army. Dr. Billings is a man who, by science and his country, and from his great erudition and knowledge, has deservedly won a high place in his profession, and his occupying the post of President of this great national Congress gives that meeting more distinction than it confers on him.

---

A CORRESPONDENT, who feels deeply that it is desirable for the good of both the public and the hospitals that these should be free to all who need their help, has sent us the report of the Newcastle Infirmary, which shows that in the one year which has elapsed since this institution was changed from a "ticket" to a "free" hospital, the workmen's contributions have risen from £332 to £1,550, which sum does not include £400, collected in 1887, but not handed to the treasurer of the infirmary till after the accounts were made up. The best comment we can offer on these facts may be given in the words of our correspondent, who has already stated his views on the subject in our columns : "An advance in workmen's contributions from £332 to nearly £2,000, with a proportionate increase of support to the other charities of the city, and every prospect of that amount being doubled as the scheme spreads this year, speaks for itself, and I think justifies me in pressing the claims of such a movement on the attention of hospital managers in London and other large towns." Many letters on the subject of workmen's contributions have appeared in che newspapers, and in one of the special supplements we pointed out that it was a privilege for our working classes to support hospitals when they are well if they wish to profit by them when they are ill. This statistical testimony from Newcastle proves that they are ready to do so when assured that the hospitals are really meant for all, and that necessity forms a sufficient claim to their benefits.

